# Assessment of Training in Health Disparities in US Internal Medicine Residency Programs

**DOI:** 10.1001/jamanetworkopen.2020.12757

**Published:** 2020-08-10

**Authors:** Denise M. Dupras, Mark L. Wieland, Andrew J. Halvorsen, Maria Maldonado, Lisa L. Willett, Linda Harris

**Affiliations:** 1 Mayo Clinic College of Medicine and Science, Rochester, Minnesota; 2 Icahn School of Medicine at Mount Sinai, Yonkers, New York; 3 University of Alabama School of Medicine, Birmingham; 4 Research Center, American College of Physicians, Philadelphia, Pennsylvania

## Abstract

**Importance:**

Health disparities continue to exist despite the call to increase education of health care practitioners. An assessment of health disparities education has not been previously studied in a national cohort.

**Objective:**

To describe and compare the curriculum on health disparities from the perspective of program directors and perceptions of training among internal medicine residents.

**Design, Setting, and Participants:**

This cross-sectional survey study used a survey of US internal medicine program directors, the 2015 Association of Program Directors in Internal Medicine annual survey, which included questions about health disparities curriculum, and a 1-time survey of US internal medicine residents that asked questions related to their training in health disparities on the American College of Physicians 2015 Internal Medicine In-Training Examination. All internal medicine program directors who were members of Association of Program Directors in Internal Medicine (368 of 396 accredited programs), and internal medicine residents who took the Internal Medicine In-Training Examination were eligible. Final analysis of the merged data set was completed in 2018.

**Exposures:**

Questions were included on the annual Association of Program Directors in Internal Medicine survey and the Internal Medicine In-Training Examination.

**Main Outcomes and Measures:**

Program directors reported on presence of health disparities curriculum, educational methods, quality, barriers and challenges to curriculum, and Clinical Learning Environment Review. Residents reported whether they received training and quality of the training in health disparities.

**Results:**

A total of 227 program directors (response rate, 61.7%) and 22 723 residents (response rate, 87.2%) responded to the surveys. A total of 90 program directors (39.6%) reported a curriculum in health disparities, but among these, only 16 program directors (17.8%) felt quality of their education was very good or excellent. In more than half of the programs (52 programs [55.9%]), outcomes of the curriculum were not measured. After merging, the combined data set included 18 883 residents from 366 APDIM member programs with 225 program director responses. Among these, 13 251 residents (70.2%) reported some training in caring for patients at risk for health disparities. Of residents who reported receiving training, 10 494 (79.2%) rated the quality as very good or excellent.

**Conclusions and Relevance:**

These findings suggest that that despite the Clinical Learning Environment Review mandate and Accreditation Council for Graduate Medical Education required competencies for training in health disparities, relatively few internal medicine programs in the US provided educational curriculum. Additionally, the existence of health disparities curricula in internal medicine training programs was not associated with resident’s perception of training or its quality.

## Key Points

**Question:**

Are internal medicine residents being trained to address health disparities, and what are their perceptions of such training?

**Findings:**

This survey study of 227 program directors and 22 723 internal medicine residents in training found that residents’ perceptions of training in health disparities and its quality were not associated with the presence of a curriculum.

**Meaning:**

These findings suggest that adding health disparities curricula in their current forms may not adequately train residents to address gaps in care.

## Teaser

This survey study examines the associations of internal medicine program directors’ perceptions of health disparities programs with those of the residents in internal medicine programs.

## Introduction

In 2002, the Institute of Medicine, now the National Academy of Medicine, produced their seminal report “Unequal Treatment: Confronting Racial and Ethnic Disparities in Healthcare,”^1^ which summarized the stark differences in health and health care outcomes between patients in minority groups vs those not in minority groups. Despite significant attention to that report, the 2018 “National Healthcare Disparities Report”^2^ found that while some disparities in health care based on race, ethnicity, socioeconomic status, disability, and in populations with special health needs were getting smaller, disparities persisted for poor and uninsured populations in all priority areas. The 2003 report^1^ prompted calls for increased education for all health care practitioners (including physicians) to begin to address these disparities.^3,4^ This charge is further reflected in the Health Quality Pathways of the Clinical Learning Environment Review (CLER)^5^ and required Accreditation Council for Graduate Medical Education (ACGME) competencies. Nevertheless, many trainees do not feel prepared to care for patient populations most at risk for health disparities.^6,7,8^

While these recommendations have existed for at least a decade,^9^ a 2014 review of health care disparities training in residency programs in the US^10^ demonstrated few published reports of graduate medical education programs that ensure that residents are competent to address disparities. The first national report of findings for the CLER program,^11^ highlighted health disparities as an area of challenge and opportunity for graduate medical education.^12^ The major findings were that the primary source of learning involved clinical experiences with patients who were at risk, mainly occurring in primary care settings and was generic, not tailored to the specific populations served by their medical center or hospital. In the second report based on residency site visits, 32.8% of residents and fellows reported their health disparities training was specific to their at-risk populations.^13^

Previous Association of Program Directors in Internal Medicine (APDIM) surveys have addressed some issues related to health disparities, including cultural competency training and quality improvement projects. In the 2012 survey, 16.6% of internal medicine programs reported the presence of a health disparities curriculum. There are studies describing educational curriculum within internal medicine residencies for special populations,^14,15,16,17^ and a recent survey of internal medicine program directors highlighted disparities related to patients with limited English proficiency in their curricula.^18^

A 2010 multi-institutional study of internal medicine residents’ health disparities education^19^ found that only 14% of residents surveyed felt confident in their knowledge of underserved populations. However, a 2011 national survey^20^ showed that residents’ attitudes toward topics regarding medically underserved populations and health disparities were generally favorable. The goals of this study were to describe what internal medicine residency training programs provide as curriculum and/or educational experiences on health disparities, to determine residents’ perception of training in health disparities, and to determine the association of program curriculum with resident perception of training.

## Methods

The survey study was reviewed and deemed exempt by the Mayo Clinic institutional review board. Program director surveys were granted a waiver of informed consent because data were deidentified, per APDIM policy. All residents who completed surveys provided written informed consent. This study is reported in accordance with the American Association for Public Opinion Research (AAPOR) reporting guideline.

### Program Director Survey

The APDIM Survey Committee develops and distributes an annual survey to internal medicine program directors to address important issues in graduate medical education. The survey process has been previously described.^21,22^ For this study, email notifications with program-specific hyperlinks to a web-based questionnaire were sent in August 2015 to 368 program director members, representing 92.9% of 396 US categorical internal medicine residencies. Reminders were sent to nonresponders biweekly, and personal contact was initiated by committee members in October. The survey closed in November 2015.

The survey collected information on characteristics of the program and program directors, as well as items on health disparities training availability, training content domains, barriers to implementation, and whether the institution had a CLER visit (eAppendix 1 in the Supplement). Survey responses were appended with data from publicly available sources, including the US Census Bureau, the American Medical Association Fellowship and Residency Electronic Interactive Database Access System Online,^23^ and the American Board of Internal Medicine.^24^ Data from the public ACGME website^25^ included accreditation cycle length, government affiliations, number of approved and filled training positions, and program director’s appointment date.

### Resident Physician Survey

Detailed information concerning the internal medicine In-Training Examination (IM-ITE) resident survey has been reported previously.^26^ Briefly, the IM-ITE is a standardized examination developed by a Committee of the American College of Physicians that serves as a self-assessment of medical knowledge for internal medicine residents. Nearly 100% of internal medicine residency programs accredited by the ACGME participate in the IM-ITE, which is administered every August through September. On completion of the IM-ITE, residents are asked to complete a voluntary survey, which is submitted with their online examination. The survey questions are designed to gain an understanding of residents’ training environment, and residents are asked for their consent to allow responses to be used in research. The 2015 survey included 3 questions related to a residents’ perception of their training in health disparities (eAppendix 2 in the Supplement).

### Comparison of Program Director and Resident Responses

Data from the program director and resident surveys were linked by training program via a unique examination identification number for each internal medicine examinee prior to being deidentified to compare program director responses with residents’ perceptions of training in health disparities. The inclusion of questions on the APDIM and IM-ITE surveys related to training in health disparities provided a unique opportunity to compare the program director–reported curriculum within their internal medicine training programs with the perceptions and experiences of the residents in training.

### Statistical Analysis

Univariate summaries of program director and resident responses were reported as count (with percentage) or mean (with SD), as appropriate. To test the association of resident report of receiving training on health disparities with the proportion of patients considered at-risk whom they cared for, a generalized linear mixed model with a binary response distribution was used to estimate odds ratios. To test the association of PD report of presence of a health disparities curriculum with resident rating of the quality of their training in care for underserved, a generalized linear mixed model with a Gaussian response distribution was used to estimate the mean difference between groups. Both models included random effects to account for nesting of residents within programs. *P* values were 2-sided, and the threshold for statistical significance was set at *P* < .01 to account for multiple comparisons. Statistical analyses were conducted using SAS statistical software version 9.4 (SAS Institute). Final analysis of the merged data set was completed in 2018.

## Results

The program-level data set included 408 training programs, including 396 ACGME internal medicine programs (97.1%), and 368 were APDIM member programs (92.9%), among which 227 program directors (61.7%) responded to the survey. The resident-level data set included 22 723 residents who completed the survey and consented to research use (response rate, 87.2%). After merging, the combined data set included 18 883 residents from 366 APDIM member programs, with 225 program director responses. For analyses requiring identifiably linked PD and resident survey responses, there were 11 583 resident responses available from these 225 responding programs. There were no differences in response rates based on geographical region or program type.

### Program Director Survey Results

Most respondents had experienced a CLER visit (160 program directors [71.0%]). Among 66 program directors who had not experienced a CLER visit, 43 (65.1%) expected one in the next 6 months. Only 57 program directors (25.0%) reported that benchmark data on patient diversity or groups at risk for health disparities was disseminated at their institutions. Slightly more than half (127 program directors [56.4%]) reported that some resident quality improvement projects addressed health disparities.

A total of 91 program directors (39.6%) reported a health disparities curriculum. While the mean (SD) time dedicated to the curriculum was 11.4 (20.9) hours, the median (interquartile range) was only 6 (4-10) hours. Nearly all programs included education about racial/ethnic diversity (84 programs [90.3%]) as well as socioeconomic status (84 programs [90.3%]). More than half of programs reported including information about limited English proficiency (54 programs [58.1%]), gender (54 programs [58.1%]), and gender identity/sexual orientation (49 programs [52.7%]). Religious beliefs were addressed in 41 programs (44.1%). Reported educational methods included lectures (66 programs [71.0%]), group discussion (50 programs [53.8%]), and clinical experiences (37 programs [39.8%]). Most program directors rated the quality of their health disparities educations as fair (35 program directors [38.9%]), good (39 program directors [42.4%]), or very good (15 program directors [16.7%]). For most of the programs (52 programs [55.9%]), outcomes of the curriculum were not measured. Assessment of the curriculum included direct observation of residents (33 programs [35.5%]), but more rarely clinical outcomes (9 programs [9.7%]), resident attitudes (8 programs [8.6%]), or knowledge (8 programs [8.6%]).

Among 132 programs that did not have a curriculum, barriers to development included time within the current curriculum (reported by 64 program directors [48.5%]), insufficient faculty skill (reported by 63 program directors [47.7%]), lack of institutional support (reported by 42 program directors [31.8%]) and lack of faculty interest (reported by 29 program directors [22.0%]). Only 40 program directors (30.5%) intended to develop and implement a curriculum within 1 year; 40 program directors (30.5%) had no plans for a curriculum, and 51 program directors (38.9%) were unsure.

### Resident Survey Results

The 18 883 resident survey responses were similarly distributed across the 3 years of training, with 6403 residents (33.9%) in postgraduate year 1, 6571 residents (34.8%) in postgraduate year 2, and 5909 (31.3%) in postgraduate year 3. Overall, 13 251 residents (70.2%) reported some training in caring for patients at risk for health disparities. This percentage did not differ significantly based on geographic location of training. The perception of receiving training increased with each additional postgraduate year.

Among 13 251 residents who reported training, more than three-quarters (10 294 residents [77.7%]) rated the quality of this training as being very good (5503 residents [41.5%]) or excellent (4791 residents [36.2%]) (Table 1). There was an association between a resident’s perceived receipt of training and their estimated proportion of patients who would be considered at risk for health disparities (Table 2).

**Table 1.** Program Director and Resident Rating of Training in Health Disparities

Rating	No. (%)	
	Program directors (n = 90)	Residents (n = 13 251)
Poor	2 (2.2)	47 (0.4)
Fair	35 (38.9)	524 (4.0)
Good	39 (42.4)	2386 (18.0)
Very good	15 (16.7)	5503 (41.5)
Excellent	1 (1.1)	4791 (36.2)

**Table 2.** Association of Resident Perception of Health Disparities Training With Proportion of Underserved Patients in Their Practice

Perceived underserved patients treated, %	Residents reporting training (n = 18 883)	
	Odds ratio (99% CI)	*P* value
<10	1 [Reference]	
10-25	1.34 (1.10-1.64)	<.001
26-50	1.74 (1.42-2.13)	<.001
51-75	2.02 (1.64-2.48)	<.001
>75	2.31 (1.86-2.87)	<.001
Not sure	0.97 (0.78-1.21)	.69

### Comparison of Program Director and Resident Responses

While less than 40% of program directors reported a curriculum for training in health disparities, most residents reported training in the care of patients who are at risk for health disparities (ie, those who are underserved, uninsured, unemployed, or experiencing homelessness). We observed no association between the program director–reported presence of a curriculum and the resident report of training or their rated quality of their training in the merged data set of 225 program director responses matched with 11 583 resident responses (Table 3).

**Table 3.** Association of Program Director–Reported Health Disparities Curriculum and Resident’s Perception of Quality of Disparities-Related Training

Presence of curriculum	Programs, No.	Resident rating of training, No. (%)						
		Excellent	Very good	Good	Fair	Poor	No training	Totals
Yes	91	1462 (27.1)	1683 (31.2)	620 (11.5)	145 (2.6)	13 (0.2)	1479 (27.3)	5402 (28.6)
No	133	1450 (23.6)	1742 (28.3)	769 (12.5)	159 (2.6)	10 (0.2)	2008 (32.7)	6138 (32.5)
No response	142	1879 (25.6)	2078 (28.3)	997 (13.6)	220 (3.0)	24 (0.3)	2145 (29.2)	7343 (38.9)
Total	366	4791 (25.4)	5503 (29.1)	2386 (12.6)	524 (2.8)	47 (0.2)	5632 (29.8)	18 883

## Discussion

This survey study is the first to our knowledge to report on the breadth of health disparities training within internal medicine training programs in the US. While approximately 70% of program directors reported a CLER visit, suggesting awareness of the requirement for health disparities training, only 40% of program directors reported having a curriculum. The curriculum was often limited to a few hours and lecture-based, which may not effectively engage learners. There was limited assessment of the impact of the curriculum on outcomes for the learner or the patients they care for. Only 30% of internal medicine programs without a current curriculum planned to address this need within the next year. While this was an improvement over the 2012 survey, in which only 16.6% of programs reported a curriculum, our findings raise concerns that additional efforts are needed for internal medicine graduate medical education to increase health disparities curricula.

Challenges to developing curriculum were identified by program directors, most significantly competing curricular priorities. There was the additional challenge of determining what a health disparities curriculum should contain (eg, cultural competency, health disparities, health inequities, social determinants of health). Unlike undergraduate medical education, time for authentic community engagement is limited during graduate medical education. This leaves didactic curricula (eg, lectures, online modules) and quality improvement work as the primary educational activities. Didactic curriculum, while efficient, does not fully foster understanding of the lived experience of health inequities nor foster cultural humility. Results of this study suggest that quality improvement initiatives aimed at reduction of practice-based health disparities are hampered by lack of access to data, that is, only 25% of program directors reported access to data that stratify according to different populations. Indeed, most published reports of curricular innovations in health disparities education in residency focus on educational outcomes rather than improvement in patient outcomes.^27,28,29,30^

To our knowledge there are no prior studies that have reported results from a national survey of internal medicine resident perceptions on training in health disparities. Overall, 70% of residents reported training. Residents who cared for a larger proportion of underserved patients perceived that they received health disparities training at a higher rate. Since an essential component of graduate medical education is direct patient care and academic medical centers provide care for a disproportionate proportion of underserved populations in the US,^31^ this may provide an opportunity for meaningful learning in the context of graduate medical education. Point of care teaching around health disparities and social determinants of health can be a powerful mechanism for residents to internalize these complex topics through the eyes of their own patients.^32^ A comment on the program director survey summarized this practice: “…we take care of the patients in our community with health care disparities. We live it every day!” However, reliance on learning in the context of caring for patients who are underserved does not ensure residents will receive the training and acquire the skills they will need. Accordingly, a 2019 study found that increased care of at-risk populations did not translate into increased relevant knowledge among resident physicians.^33^ Additionally, while some data suggest a positive influence of care of underserved populations on the attitudes of residents, there was no improvement in patient care with additional training and an inverse association was found between self-reported skills and knowledge.^34^ Our finding that residents’ rating of the quality of their training was not associated with the presence of a curriculum in health disparities in their program also raises a concern that perceptions may overestimate the acquisition of needed skills.

Strengths of this study include a large, representative, and comprehensive sample of all internal medicine residents and program directors in the US with high survey response rates. Furthermore, the ability to link responses of residents with their programs through coincident surveys in the same calendar year provided a powerful tool for comparison of resident perceptions and curriculum.

### Limitations

The study has limitations. A major limitation was that residents were not asked directly if they were exposed to a curriculum in health disparities but rather if they received training in the care of patients who would be at risk, which raises the concern that we cannot distinguish between their recognition of a formal and informal curriculum. Additionally, while program directors were asked explicitly whether they had a curriculum, we cannot know with certainty that they were aware of all training, but given their reporting responsibilities, we feel it is a reasonable assumption. Furthermore, because the survey items were embedded in larger program director survey, we were limited in the ability to ask them to define more specifically the components of their health disparities curricula.

## Conclusions

This survey study found that the existence of health disparities curricula among internal medicine residency programs were below goal levels established by national accrediting bodies and that existing curricula were not associated with resident’s perception of training in these domains. Residents who cared for more patients who are underserved reported higher disparities training despite a lack of formal curricula, highlighting the opportunity to teach around health disparities at the point of care, but also the need for standardized curriculum and capable faculty. Additionally, program directors reported a lack of practice-level data to examine health disparities, which emphasizes the opportunity for institutional collaboration with residency programs for quality improvement initiatives aimed at the reduction of health disparities.

Future research is needed to develop and assess the most relevant domains for health disparities curricula in graduate medical education, including for point-of-care encounters and quality improvement. There are opportunities to explore partnerships among residencies, institutional clinical practices, and communities for productive collaborations around disparities-related quality improvement projects to address gaps in health care that are specific to the populations they serve.

## Notes

Supplement. **eAppendix 1**. Association of Program Directors in Internal Medicine 2015 Annual Survey **eAppendix 2.** Questions on the 2015 Internal Medicine American College of Physicians In-Training Examination
