# Supplementary material for: Assessment of Training in Health Disparities in US Internal Medicine Residency Programs
Source: JAMA Netw Open. 2020 Aug 10;3(8):e2012757. doi: 10.1001/jamanetworkopen.2020.12757 (PMC7417967; doi:10.1001/jamanetworkopen.2020.12757)
Supplement: Supplement. — eAppendix 1. Association of Program Directors in Internal Medicine 2015 Annual Survey eAppendix 2. Questions on the 2015 Internal Medicine American College of Physicians In-Training Examination [file jamanetwopen-3-e2012757-s001.pdf]

Supplementary Online Content

Dupras DM, Wieland ML, Halvorsen AJ, Maldonado M, Willet LL, Harris L. Assessment of training in health disparities in US internal medicine residency programs. *JAMA Netw Open*. 2020;3(8):e2012757. doi:10.1001/jamanetworkopen.2020.12757

eAppendix 1. Association of Program Directors in Internal Medicine 2015 Annual Survey
eAppendix 2. Questions on the 2015 Internal Medicine American College of Physicians In-Training Examination

This supplementary material has been provided by the authors to give readers additional information about their work.

© 2020 Dupras DM et al. *JAMA Network Open.*

eAppendix 1. Association of Program Directors in Internal Medicine 2015 Annual Survey

SECTION V: Health Care Disparities Training (Please ENTER ALL NUMBERS WITHOUT COMMAS to avoid truncation at the comma.)

Has your Institution had a Clinical Learning Environment Review (CLER) by the ACGME visit?
- ❍ No
- ❍ Yes

Do you anticipate one in the next 6 months?
- ❍ No
- ❍ Yes

Is data on differences in health care benchmarks based on patient diversity or at risk populations disseminated at your institution?
- ❍ No
- ❍ Yes
- ❍ Don't know

How committed do you believe your institution is to addressing disparities in health care?
- ❍ Not committed
- ❍ Unsure
- ❍ Somewhat committed
- ❍ Strongly committed

Do some of your resident quality improvement projects address health care disparities?
- ❍ No
- ❍ Yes
- ❍ I don't know

Branch point question: Answer selected determines subsequent questions
Does your training program have an educational curriculum in health care disparities?
- ❍ No
- ❍ Yes

Following questions answered by those who answered "yes" to above question:
What were barriers to development of a health disparities curriculum? (Mark all that apply.)

- ❑ No barriers
- ❑ Institutional culture
- ❑ Institutional support
- ❑ Lack of need
- ❑ Faculty uninterested in teaching
- ❑ Faculty lack skills to teach
- ❑ Lack of learner interest
- ❑ Insufficient time in curriculum
- ❑ Financial constraints
- ❑ Lack of importance in the curriculum
- ❑ Other, please specify: ____________________

What were barriers to implementation of a health disparities curriculum? (Mark all that apply.)

- ❑ No barriers
- ❑ Institutional culture
- ❑ Institutional support
- ❑ Lack of need
- ❑ Faculty uninterested in teaching
- ❑ Faculty lack skills to teach
- ❑ Lack of learner interest
- ❑ Insufficient time in curriculum
- ❑ Financial constraints
- ❑ Lack of importance in the curriculum
- ❑ Other, please specify: ____________________

<u>Please answer the following questions about the curriculum.</u>

Which of the following best describes the format of the curriculum?

- ❍ Required by the Institution
- ❍ Only required by residency training program
- ❍ Elective for residents
- ❍ Component of other curriculum (quality improvement, other)

What curriculum do you use?

- ❍ Developed at our Institution
- ❍ External curriculum. Please provide name of program below: ____________________

How many hours in your program are dedicated education in health care disparities? ____

© 2020 Dupras DM et al. *JAMA Network Open.*

Does the curriculum vary between years?
❍ No
❍ Yes

What methods are used for this education? (Mark all that apply.)
❑ Lecture
❑ Discussion (group)
❑ Small groups
❑ Case scenarios
❑ Clinical experience
❑ Interviewing other cultures (at risk groups)
❑ eLearning
❑ Blogs
❑ Audio/video
❑ Simulation
❑ Role play
❑ Cultural immersion
❑ Other, please specify: ____________________

Which topics related to disparities of care are covered in your educational curriculum? (Mark all that apply.)
❑ Race and/or ethnicity
❑ Gender
❑ Socioeconomic status
❑ Sexual orientation and/or gender identity
❑ Religious beliefs
❑ Limited English proficiency
❑ Other, please specify: ____________________

How would you rate the quality of your program's health disparities education?
❍ Poor
❍ Fair
❍ Good
❍ Very good
❍ Excellent

How do you measure outcomes of the curriculum (Mark all that apply.)
❑ We don't measure
❑ Assessment of change in attitude(s)
❑ Test of knowledge
❑ Direct observation of resident skills/behaviors
❑ Clinical patient outcomes
❑ Other, please specify: ____________________

© 2020 Dupras DM et al. *JAMA Network Open.*

Following questions answered by those answering “no” to presence of curriculum:
What are barriers to development of a health disparities curriculum? (Mark all that apply.)

- ❑ No barriers
- ❑ Institutional culture
- ❑ Institutional support
- ❑ Lack of need
- ❑ Faculty uninterested in teaching
- ❑ Faculty lack skills to teach
- ❑ Lack of learner interest
- ❑ Insufficient time in curriculum
- ❑ Financial constraints
- ❑ Lack of importance in the curriculum
- ❑ Other, please specify: ____________________

Do you plan to develop education curriculum on health care disparities?

- ❍ No
- ❍ Yes
- ❍ I don't know

How soon do you plan to implement an educational curriculum on this topic?
Number of ___ days ___ months ___ years

End of survey question section.

© 2020 Dupras DM et al. *JAMA Network Open.*

eAppendix 2. Questions on the 2015 Internal Medicine American College of Physicians In-Training Examination

1. Approximately what percentage of the patients you care for is underserved, uninsured, unemployed or homeless? (Select one)

__ <10%
__ 10-25%
__ 26-50%
__ 51-75%
__ Over 75%
__ Not Sure

2. During your residency, have you received any training in how to care for patients who are underserved, uninsured, unemployed or homeless?

__ Yes
__ No

3. How would you rate the quality of this residency training? (Likert Scale Response)

__ 1 Poor
__ 2 Fair
__ 3 Good
__ 4 Very Good
__ 5 Excellent

© 2020 Dupras DM et al. *JAMA Network Open.*
